# Performance Assessment of ChatGPT versus Bard in Detecting Alzheimer’s Dementia

**DOI:** 10.3390/diagnostics14080817

**Published:** 2024-04-15

**Authors:** Balamurali B.T, Jer-Ming Chen

**Affiliations:** Science, Mathematics & Technology (SMT), Singapore University of Technology & Design, 8 Somapah Rd, Singapore 487372, Singapore

**Keywords:** Large Language Models, chatbots, GPT-3.5, GPT-4, ChatGPT, Bard, Alzheimer’s dementia, zero-shot learning, chain-of-thought, ecological diagnostic screening, spontaneous speech

## Abstract

Large language models (LLMs) find increasing applications in many fields. Here, three LLM chatbots (ChatGPT-3.5, ChatGPT-4, and Bard) are assessed in their current form, as publicly available, for their ability to recognize Alzheimer’s dementia (AD) and Cognitively Normal (CN) individuals using textual input derived from spontaneous speech recordings. A zero-shot learning approach is used at two levels of independent queries, with the second query (chain-of-thought prompting) eliciting more detailed information than the first. Each LLM chatbot’s performance is evaluated on the prediction generated in terms of accuracy, sensitivity, specificity, precision, and F1 score. LLM chatbots generated a three-class outcome (“AD”, “CN”, or “Unsure”). When positively identifying AD, Bard produced the highest true-positives (89% recall) and highest F1 score (71%), but tended to misidentify CN as AD, with high confidence (low “Unsure” rates); for positively identifying CN, GPT-4 resulted in the highest true-negatives at 56% and highest F1 score (62%), adopting a diplomatic stance (moderate “Unsure” rates). Overall, the three LLM chatbots can identify AD vs. CN, surpassing chance-levels, but do not currently satisfy the requirements for clinical application.

## 1. Introduction

Alzheimer’s dementia (AD), a progressive neurodegenerative disorder, is a growing global concern [1,2]. It significantly impacts patients’ quality of life and imposes a substantial burden on healthcare systems. Projections suggest that by 2030, the cost of Alzheimer’s care could soar up to USD 4.7 trillion globally, from USD 2.8 trillion in 2018 [3]. Therefore, early screening and comprehensive understanding of Alzheimer’s progression are paramount. Studies indicate that early interventions like lifestyle changes and social engagement could potentially slow down dementia progression, highlighting the criticality of early detection [4,5]. Despite pharmaceutical treatments being available, accurately assessing cognitive decline is imperative before initiating any intervention to mitigate disease advancement [6,7]. The deterioration of language and speech abilities often precedes other cognitive impairments in AD, making the analysis of spontaneous speech a potentially valuable tool for early AD detection [8,9]. Speech characteristics, including fluency, vocabulary usage, and vocal quality, are demonstrably affected by the underlying cognitive decline. By analysing acoustic features defining such speech patterns, researchers are exploring the possibility of developing a non-invasive, objective, and cost-effective method for predicting cognitive decline [10,11,12,13,14,15,16,17]. Furthermore, with the recent advancements in natural language processing (NLP), researchers can now transcribe speech to text and analyze the resulting text to predict cognitive decline [18,19,20,21,22]. This approach utilizes text features of varying granularities, and studies have shown promising results in detecting AD. Such automated approaches, combining acoustic and text analysis powered by machine learning [23,24], have the potential to improve patient outcomes by facilitating early AD intervention and enabling the development of targeted therapies.

Large language model (LLM) chatbots such as OpenAI’s Generative Pretrained Transformer (GPT, versions 3.5 and 4) and Google’s Bard, demonstrate impressive capabilities in many domains, including healthcare settings [25,26,27], to support early detection and clinical assessment. Here, we explore the utility of LLM chatbots (ChatGPT-3.5, ChatGPT-4, and Bard) for identifying AD in individuals, using textual transcriptions derived from spontaneous speech: a non-trivial assessment task that currently poses significant challenges for other state-of-the-art detection modalities and could benefit immediately from advanced artificial intelligence techniques directly applied “in-the-field”.

In our recent work [10], we adopted a multi-modal approach that integrated audio- and text-based techniques to automatically detect AD from audio recordings of spontaneous speech. Notably, our text-based classification method exhibited superior performance (88.7% accuracy) compared to our audio-based classification (78.9% accuracy). At the time of reporting, this state-of-the-art accuracy surpasses other contemporaneous studies utilizing the same dataset [11,12,28,29], highlighting the efficacy of the text-based approach in reliably identifying AD. Motivated by this, we investigate here the potential viability of LLM chatbots—in their ‘as-is’ form (i.e., utilizing zero-shot learning in which the model is expected to make predictions about classes it has not been explicitly trained on [30,31])—as a possible tool to detect AD using only spontaneous speech. While previous studies using audio, text, or their fusion with model embeddings as features achieved promising AD/CN classification accuracies ranging from 78 to 88% [10,11,12,24,28,29,32,33,34], they all relied on supervised learning procedures with labelled training data. This limits their generalizability to real-world settings lacking readily available labels. In contrast, the zero-shot LLM chatbot learning approach in the current investigation assesses the feasibility of using chatbots in-the-field without requiring labelled training data. Therefore, direct comparisons of LLM chatbots’ performance with existing results from supervised models would be inappropriate. Instead, our investigation focuses on evaluating the appropriateness and clinical efficacy of LLM chatbot responses as a supplementary framework for AD assessment.

The manuscript is structured as follows: Section 2 provides a comprehensive overview of the methodology followed in this investigation, detailing the approach taken to pre-process the audio data, convert it to text, prompt the LLM chatbots, and obtain the chatbots’ responses. The results of this investigation are presented in Section 3, where key findings and observations about the LLM chatbots’ responses, its prediction performance, its chains of thought, and the correlation between chatbots’ responses and Mini-Mental State Examination (MMSE) scores are analyzed in depth. Section 4 presents discussion of the results, exploring the implications of this new approach for AD detection using LLM chatbots and its significance in the broader context of the study. Additionally, it discusses the drawbacks of this approach. Finally, Section 5 concludes the report, summarizing the key findings of these chatbots’ AD predictions and suggesting avenues for future research and exploration.

## 2. Methods

We survey three state-of-the-art LLM chatbots: OpenAI’s language models GPT-3.5 and GPT-4 (14 March 2023 ChatGPT version; http://openai.com) [35] and Google’s language model, Bard (10 May 2023 version; https://bard.google.com) [36]. Throughout this paper, ‘GPT-3.5’ and ‘GPT-4’ refer to OpenAI’s corresponding ChatGPT 3.5 and ChatGPT 4 generative pre-trained transformer chatbots. To investigate their ability to identify subjects with AD compared to Cognitively Normal (CN) subjects based on the input text, we employed a zero-shot learning approach, where LLM chatbots were presented with the transcribed text as a single input at two levels of independent prompts, with the second query being more detailed than the first:

- Query 1 (Q1). “Could the following transcribed speech be from a Cognitive Normal or Alzheimer’s Dementia subject?”
- Query 2 (Q2). “Can you look at the syntax, vocabulary, structure, narration style, grammar, semantic discourse, stylistics, pragmatics and share your opinion in short concise points on what you think of the following paragraphs? These paragraphs are transcribed text from an interview with different subjects. Could they be narrated by a Cognitive Normal or an Alzheimer’s Dementia subject?”

A less structured chain-of-thought prompting approach, a strategy which is different from step-by-step thinking [31], was followed in this investigation. Also, in the context of this study, the prompts used to query the chatbots are independent, and as a result, the benefit commonly associated with information extraction strategies [37], transforming zero-shot into multi-turn question answering [38], cannot be availed of. Although, these strategies could be included in the future and are likely to improve the performance, this study specifically focused simply on the feasibility and utility of LLM chatbot’s for identifying AD from text transcribed from speech audio.

A well-studied dataset of audio recordings provided in the ADReSSo Challenge [39] was utilized in this study. Specifically, these audio recordings captured interview sessions where participants described the “Cookie Theft Picture” from the Boston Diagnostic Aphasia Examination [40] in English. Audio segments containing the interviewer’s speech, including any overlap with the subject, were removed, while retaining non-speech segments such as silence and filler words. To facilitate textual input, the speech audio was transcribed into text using the Otter.ai platform [41]. Given the zero-shot nature of our approach, we focused on the 71 recordings (36 CN, 35 AD) constituting the testing set of ADReSSo Challenge [39], aligning with our prior work [10] for comparison and generalizability assessment.

**Figure 1:**
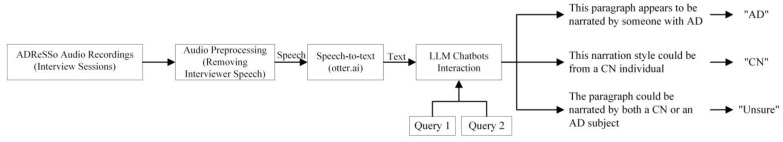
Experimental methodology illustrating LLM chatbot zero-shot learning for predicting the ADReSSo speech recordings (71 testing set recordings 36 CN, 35 AD) as “AD”, “CN”, and “Unsure”.

Text from each recording, accompanied with the queries (Q1 and Q2), was presented as an input prompt only once to each LLM chatbot. The corresponding output responses generated by each LLM chatbot, with typical phrases such as “This paragraph appears to be narrated by someone with AD”, “This narration style could be from a CN individual”, or “The paragraph could be narrated by both a CN or an AD subject”, were then classified accordingly as “AD”, “CN”, or “Unsure” categories (See Figure 1, the experimental methodology depicting LLM chatbots zero-shot learning approach to predicting speech recordings as ‘AD’, ‘CN’, and ‘Unsure’). The relationship between predicted LLM chatbots outcomes for each recording and subjects’ Mini-Mental State Examination (MMSE) scores was examined to identify any specific trends or patterns.

A note on repeatability: Although LLM chatbots are known to evolve over time as they dynamically learn and update their knowledge continuously, our preliminary investigation on repeatability revealed that the classification outcomes made within a week remained largely consistent (within 10% consistent). This implies a level of stability in the predictions made by LLM chatbots, at least within the investigative timeframe (GPTs: week of 13 March 2023; Bard: week of 10 May 2023). Upon querying the temperature parameter (the parameter that controls the randomness and creativity, a higher temperature results in creative, diverse responses, but results in factual error, while a lower temperature creates conservative, less-engaging responses and are usually accurate) used for prompt replies, ChatGPT reported a fixed value set around 0.7. In contrast, Bard’s temperature setting is characterized by its dynamic nature, adapting to the specific requirements of each prompt and context [42].

## 3. Results

The results of this investigation are divided into the following four sections: (1) the outcome matrices (True vs. Predicted classes) for Query 1 and Query 2 are summarized and compared for three LLM chatbots; (2) the performance metrics of each LLM chatbot targeting AD and CN classes are then scored separately; (3) the chain-of-thought prompting is analyzed (Query 2), offering insight into linguistic motivations; and (4) LLM chatbots’ prediction outcomes are compared against the subject’s Mini-Mental State Examination (MMSE) scores.

### 3.1. Performance and Outcomes from Prompts (Q1 and Q2)

**Figure 2:**
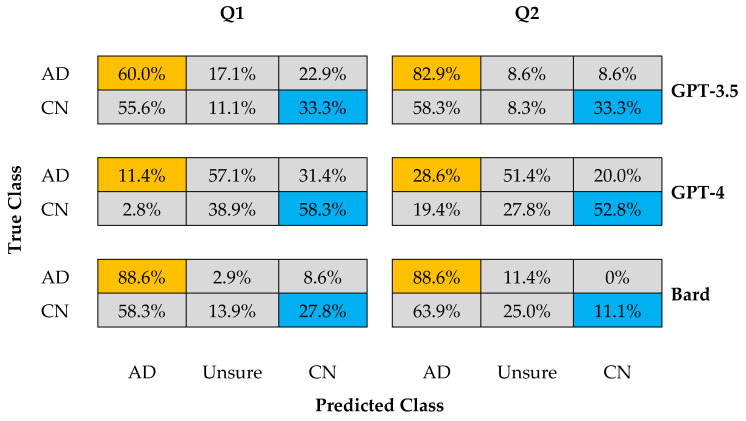
Summary matrices (True vs. Predicted) of three LLM chatbots studied for Query 1 (Q1) and Query 2 (Q2), showing cognitive classification outcomes for three class prediction (AD vs. CN vs. Unsure) and their occurrence rate (%) when presented with the same text dataset from AD and CN subjects (True Class, 35 and 36 subjects, respectively).

In response to prompts Q1 and Q2, the output generated by all three LLM chatbots fell into three categories: AD, CN, and an additional category of “Unsure” (see confusion matrices in Figure 2) (For a three-class outcome, naively considering that zero-shot outcomes arise randomly with equal probability, the chance level is arguably 33%. While our zero-shot queries yielded three-class outcomes (as would be typically encountered) despite eliciting binary ground truths, expecting the two-class outcome at 50% chance level (typical in supervised learning settings) becomes inappropriate: firstly, it disregards the full spectrum of nuanced responses captured by chatbots in this zero-shot context; secondly, it introduces bias by forcing data into a pre-defined, binary framework, which does not capture the chatbot’s true behavior). The appearance of the “Unsure” category indicates that in some instances LLM chatbots faced challenges in classifying AD vs. CN confidently and equivocated on the cognitive status of certain subjects. This uncertainty arises unsurprisingly (in hindsight), as it reflects the complexity and variability of linguistic patterns in spontaneous speech, as well as limitations of a zero-shot learning approach. It also signals agency on the LLM chatbots’ part to offer a third option despite a binary choice being clearly solicited.

GPT-3.5 demonstrated good performance in correctly detecting AD subjects, with accuracy of 60% and 83% for prompts Q1 and Q2, respectively. However, its performance in correctly identifying CN subjects was only moderate, with 33% accuracy (chance level) for both prompts. Notably, GPT-3.5 exhibited a pronounced tendency to misclassify CN subjects as AD, with high misidentification rates of 56% and 58% for the two prompts, respectively. GPT-3.5 generally displayed confidence in its predictions, as indicated by the relatively low rates of “Unsure” responses (17% and 9% for AD subjects; 11% and 8% for CN subjects, across the two prompts, respectively).

GPT-4, on the other hand, excelled in correctly detecting CN subjects, achieving accuracies of 58% and 53% for Q1 and Q2 prompts, respectively. However, its performance in correctly identifying AD subjects was poor, with accuracies of 11% and 29% for the two prompts. GPT-4 displayed a moderate tendency to misidentify AD subjects as CN, with misidentification rates of 31% and 20% for the two prompts, respectively. Notably, the model exhibited a ‘diplomatic’ response pattern where it equivocated with the highest rates of “Unsure” responses (57% and 51% for AD subjects; 39% and 28% for CN subjects, across the two prompts, respectively), hesitant to commit to a prediction between AD and CN, while showing greater uncertainty for AD subjects than CN subjects.

Bard demonstrated the strongest performance in correctly detecting AD subjects, achieving 89% accuracy for both Q1 and Q2 prompts. However, it was the poorest among the three LLM chatbots at identifying CN subjects correctly, with accuracies of 28% and 11% for the two prompts, respectively. Bard also displayed a strong tendency to misclassify CN subjects as AD, with misidentification rates of 58% and 64% for the two prompts, respectively. Like GPT-3.5 (and unlike GPT-4), Bard exhibited confidence in its predictions, as reflected by low rates of “Unsure” responses (2% and 11% for AD subjects; 14% and 25% for CN subjects, across the two prompts, respectively).

The three LLM chatbots show fair consistency regarding the Q1 and Q2 prompts, indicating at a gross level that a models’ performance is not sensitive to how the query is structured per se—a straightforward query suffices, if no further supporting details or insight is sought. Both Bard and GPT-3.5 performed similarly (and better) at correctly detecting AD subjects compared to GPT-4; in contrast, GPT-4 performed better at identifying CN subjects, while also preferring ‘diplomatic’ responses, contributing to the “Unsure” prediction class.

### 3.2. Performance Metrics of Three LLM Chatbots for Q1 and Q2

Traditional binary classification metrics, such as sensitivity, specificity, and precision, are readily computable but become problematic for our investigation. All three LLM chatbots that were queried yielded three potential outputs (AD, CN, and “Unsure”) despite the binary classification problem (AD vs. CN). Several approaches could address this mismatched output response: (1) Forced choice prompts—LLM chatbots could be prompted to explicitly yield AD or CN outputs, even if the chatbot is uncertain. This can be achieved by incorporating additional instructions within the prompts. (2) Binning “Unsure” as AD—considering all “unsure” outputs as AD (reflecting the clinical practice that “Unsure” cases are flagged for further investigation). In this study, however, we chose to analyze the performance of LLM chatbots separately when predicting for AD class and when predicting for CN class, as we recognize these two prediction tasks are neither complementary nor symmetric; accordingly, this nuanced approach provides better insight into the respective strengths/weaknesses, biases, and transparency of each LLM chatbot.

Specifically, when focusing on predicting AD (true class), the complement duly consists of the predicted “Unsure” and CN classes. Conversely, when focusing on predicting CN (true class), the complement accordingly comprises the “Unsure” and AD classes. Table 1 summarizes the performance metrics (accuracy, sensitivity, specificity, precision, and F1 score) derived for the three LLM chatbots in response to the Q1 and Q2 prompts in Figure 2.

Comparing Q1 and Q2 prompts reveals that, on average, Q2 elicits better performance for most metrics in GPT-3.5 and GPT-4, but not for Bard.

When comparing the performance of predicting AD versus predicting CN, the aggregated overall mean of the performance metrics of the three LLM chatbots for the two queries together suggests that predicting CN (56%) is slightly better than predicting AD (54%); both tasks exhibit relatively comparable performance, regardless of model. GPT-3.5 and Bard perform similarly well in predicting both CN and AD, achieving 57% and 58% for predicting CN, and 56% and 59% for predicting AD, respectively and on average. On the other hand, GPT-4 performs relatively better at predicting CN (average of 54%) compared to predicting AD (average of 48%). Notably, both GPT-3.5 and Bard outperform GPT-4 overall.

Depending on the task, certain performance metrics can be exceptionally high. For instance, when predicting CN, Bard achieves 100% specificity and precision. Similarly, when predicting AD, GPT-4 achieves 95% specificity, while Bard achieves 89% sensitivity. Additionally, predicting CN tends to exhibit more extreme performance metrics ranging from 36% to 76%, compared to predicting AD, which ranges from 48% to 61%.

Because of the presence of the “Unsure” class arising, the performance metrics of the three LLM chatbots vary depending on whether the focus is to identify AD subjects or to identify CN subjects, except for the accuracy metric (identical for both objectives).

In terms of overall accuracy (considering the average of both Q1 + Q2 outcomes together), GPT-3.5 and Bard again performed comparably, with scores averaging 52% and 54%, respectively. However, GPT-4 had the lowest overall accuracy at 38% (just above chance level). Bard exhibited the highest sensitivity (89%) when predicting AD (averaged for Q1 + Q2), while GPT-4 showed the highest sensitivity (56%) when predicting CN. GPT-4 demonstrated the highest specificity (84%) and precision (70%) when predicting AD (averaged for Q1 + Q2), while Bard achieved the highest specificity (96%) and precision (89%) when predicting CN.

The F1 score, which indicates how precision and sensitivity balance out at the expense of the other (with the proviso that both measures are equally important), reveals that Bard robustly outperforms the other LLM chatbots in predicting AD (achieving the highest F1 score of 71%, averaged for Q1 + Q2); GPT-4, on the other hand, shows the lowest F1 score at 29% (averaged for Q1 + Q2). Interestingly, the reverse occurs when predicting CN: GPT-4 achieved the highest F1 score of 62% (averaged for Q1 + Q2), while Bard showed the lowest F1 score at 31% (averaged for Q1 + Q2).

These findings underscore the understanding that predicting for AD and predicting for CN are disparate objectives, with challenges which are not simply complementary nor commensurate. Accordingly, the LLM chatbots respond differently in their capacity to correctly distinguish between AD and CN. However, from a healthcare standpoint, it is essential to highlight that an LLM chatbots-based approach is capable of achieving sensitivity exceeding 58%, as demonstrated by GPT-4 for Q1 (the most robust among all query and LLM chatbots) for classifying CNs into a control group, and thus holds promise. This emphasis is vital, considering approaches that misdiagnose a notable portion of healthy individuals as having dementia, potentially resulting in distressing false-positive outcomes.

### 3.3. Insights from Chain-of-Thought Prompting (Query 2)

**Figure 3:**
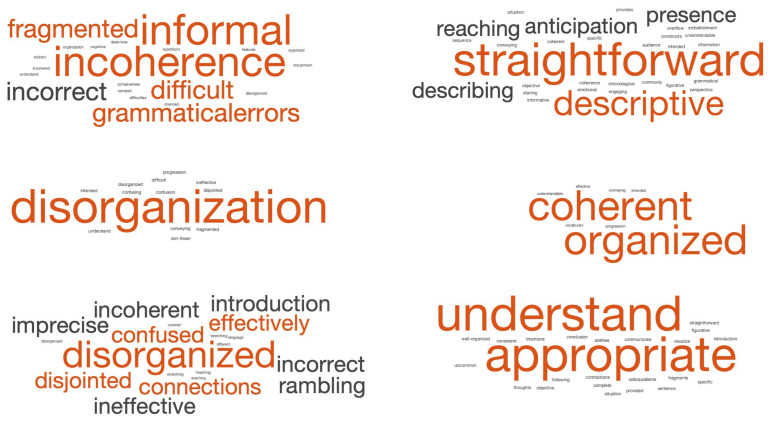
Word cloud summaries for correct AD prediction (left) and correct CN prediction (right) text output generated by GPT-3.5 (top), GPT-4 (middle) and Bard (bottom), for two AD subjects and two CN subjects.

Query 2 solicits the LLM chatbots’ chain-of-thought and reveals intermediate insights (linguistic features such as syntax, vocabulary, structure, narration style, grammar, semantic discourse, stylistics, and pragmatics) that drive its classification decisions. Accordingly, Figure 3 depicts the Query 2 output responses—aggregated and visualized as word clouds—for two AD and two CN subjects who were consistently classified correctly by all three LLM chatbots. These word clouds provide a visual representation of the prominent linguistic attributes and patterns identified by LLM chatbots, in terms of word (semantic) classes and frequency of occurrence in the LLM chatbots’ output response.

Word clouds associated with AD predictions demonstrate a greater visual spread of descriptor words and are characterized by linguistic attributes such as “incoherence”, “disorganization”, “fragmented”, and “disjointed”. These findings align with known linguistic observations describing AD [43], where subjects often exhibit difficulty maintaining coherence and producing organized speech. In contrast, CN word clouds appear visually sparser, focusing heavily on two or three main keywords, and are associated with attributes such as “coherent”, “straightforward”, and “organized”. This suggests that LLM chatbots are likely relying on higher-order linguistic features to differentiate between AD and CN subjects. Figure 3 visually summarizes the distinct linguistic training and patterns utilized by each LLM chatbot for the classification task.

### 3.4. Insights from MMSE Score Comparison

**Figure 4:**
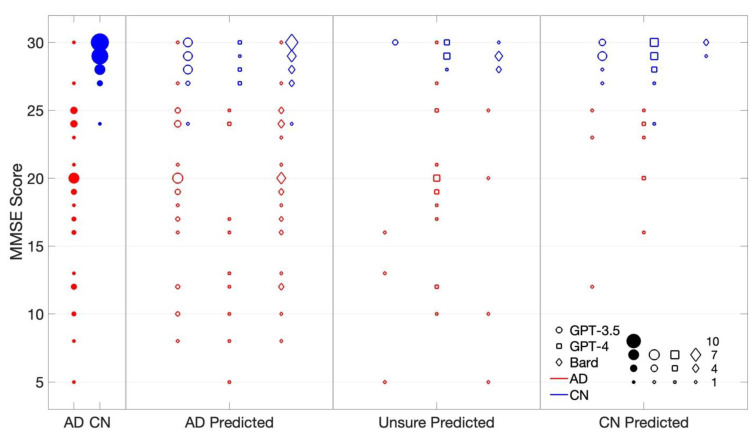
MMSE Score vs. prediction classes (AD/CN/Unsure) of Q2 for AD (red) and CN (blue) subjects across three LLM chatbots (circle: GPT-3.5; square: GPT-4; diamond: Bard); the leftmost filled circles depict MMSE score distribution for all AD and CN subjects (true class). The size of the symbols indicates the relative frequency of occurrence of that MMSE value (legend, bottom right).

The Mini–Mental State Examination (MMSE) is a common measure of cognitive impairment, where the maximum score is 30 (normal cognitive function) while scores below 23 or 24 indicate possible cognitive decline [44,45], though sociocultural variables such as age and education could affect individual scores [46]. Accordingly, Figure 4 plots MMSE scores for all subjects against the prediction response (AD/Unsure/CN) classed by the three LLM chatbots, alongside the score distribution for all AD and CN subjects. Note: two AD subjects possess MMSE scores exceeding 25 (and one CN subject scored at 24), suggesting a degree of heterogeneity within both groups.

Regardless of the prediction class (AD/Unsure/CN), there does not appear to be a clear relationship between MMSE scores and the LLM chatbots’ prediction performance for CN subjects (high MMSE scores are inherently limited in distribution). However, a significant subset of CN subjects with high MMSE scores (27–30) tend to be misclassified as AD or Unsure by all three LLM chatbots, suggesting that high MMSE scores do not necessarily aid CN prediction; other factors, such as linguistic attributes or contextual cues, may offer a confounding impact on the LLM chatbots’ prediction.

Among AD subjects, GPT-3.5 demonstrates a broad range of MMSE scores (8–30) for correctly classified AD subjects yet predicts “Unsure” even for subjects with lower scores (5–16). Additionally, it occasionally misclassifies AD subjects as CN for slightly higher MMSE scores (12–25), which may be deemed reasonable. Similarly, GPT-4 exhibits a wide distribution of MMSE scores (5–25) for correctly classified AD subjects, with scores tending to be slightly lower than those associated with “Unsure” predictions (10–30) and CN predictions (16–25). In contrast, Bard avoids predicting CN for AD subjects altogether but displays a diverse range of MMSE scores (8–30) for accurate AD classification, a trend rather like GPT-3.5’s prediction (including frequency). Notably, all three models demonstrate substantial variation in MMSE scores for correctly classified AD subjects, highlighting the tenuous relationship between MMSE scores and LLM chatbot predictions, and the limits of its utility to enhance prediction accuracy.

While the absence of a low MMSE score may lead to misclassifications of AD subjects as CN, the opposite does not hold true—high-scoring AD subjects are still correctly classified by the LLM chatbots. Overall, there does not appear to be a strong correlation between MMSE score and prediction performance for either AD or CN subjects. This is not unexpected, as the cognitive acuity of an individual varies at different instances across time, whereas the subject’s speech-based performance assessed here on a single picture-description task represents only a snapshot in time.

## 4. Discussion

Our study assessed the use of LLM chatbots for distinguishing between AD and CN classifications based on text transcriptions and provided insights into the challenges of each task and the suitability of each LLM chatbot for that objective. For positively identifying CN, GPT-4 emerges as the preferred choice, surpassing chance-level performance (true-negative at 56%). However, it should be noted that GPT-4 tends to adopt a diplomatic stance without committing to a clear prediction between AD and CN. Bard, on the other hand, stands out for positively identifying AD with an 88.6% true-positive rate. However, a limitation is observed in its tendency to misidentify CN as AD, often with high confidence. In terms of overall performance metrics, both GPT-3.5 and Bard demonstrate comparable performance in positively identifying CN.

Existing approaches for AD and CN classification employ audio, text, or their fusion, achieving accuracies of 78.9% (audio alone), 84.5% (text alone), and a range of 80.2 to 88.7% with various deep neural network models applied to fusion strategies [10,11,12,24,28,29,32,33,34]. Notably, these reported models rely on supervised learning with labelled training data. In contrast, the performance of LLM chatbots in this study does not match the level achieved by the aforementioned supervised models. However, making direct comparisons between the performance of LLM chatbots and the reported supervised models would be inappropriate due to significant differences in their underlying learning paradigms.

Why does predicting AD result in a more ‘homogenous’ performance (*cf*. Table 1) than predicting CN? First, because subjects with AD typically exhibit distinct linguistic impairments, including incoherence, disorganized speech, fragmented language, and disjointed narratives, these attributes associated with AD are often more distinguishable, leading to greater consistency and identifiability for the LLM chatbots in recognizing and predicting AD. In contrast, CN subjects span a spectrum of linguistic styles, resulting in more subtle differences in language use and a narrower range of standard observable linguistic markers compared to AD. These factors contribute to the complexity of predicting CN, as the wide variability in language patterns and the absence of clear markers make accurate CN identification challenging. Further, a majority of LLM training datasets likely consist of functional texts reflecting standard linguistic features drawing upon a large feature space encompassing broad narrative, persuasive, expository, and descriptive examples; these contrast sharply with deviations from the feature space associated with linguistic impairments arising from AD (likely also drawing on more modest datasets). Two obfuscating outcomes arise accordingly: training data imbalance, and consequently, identifying CN becomes less straightforward than identifying AD.

Among the three LLM chatbots considered, Bard exhibits a notable advantage over the GPTs in predicting AD: Bard’s responses exhibit a higher degree of detail for Q2, because it systematically considers sentence-level, paragraph-level, text-level, and discourse-level breakdowns. This richer granularity, combined with its ability to identify deviations in the trained feature space, likely contributes to Bard’s superior AD detection capabilities.

In the context of zero-shot learning, where models are not specifically fine-tuned or primed for the classification task, the direct impact of articulating the chain-of-thought response on accurate AD vs. CN classification may be expected to be limited, as zero-shot models rely on general language patterns learned during pre-training and lack explicit knowledge of task-specific labels. In our investigation, we nevertheless observed that articulating chain-of-thought responses did indeed have a positive effect on the overall performance of GPT models (but did not benefit Bard).

This study has several limitations:

1. Efficacy and Limitations of Prompts: Only two prompts (Q1 and Q2) were investigated here. It is worth considering whether more sophisticated prompting approaches would yield different outcomes. To necessitate the integration of such chatbots into healthcare practice further refinement through the formulation of more specific queries that can provide a deeper understanding of common language impairments is essential [47].
2. “Snapshot” and Limited Probing: The potential variations of outcomes across different accounts, machines, and repetitions at different times were not explored—although, as we noted during preliminary explorations of repeated prompting, using the same text for a particular subject does elicit slight variations in textual response (the extent, consistency or variation of these differences was not studied nor quantified) but not enough to influence the overall prediction outcome.
3. Non-repeatability and Dynamic Evolution of LLM chatbots: The efficacy and evolution of the LLM chatbots’ “personality” resulting from continuous querying and intervention (‘fine-tuning’) of service operators, including back-end updates to new versions, remain unknown and warrant further exploration. Performance differences between GPT-3.5 and GPT-4 is a stark example of this concern.
4. Accuracy of Transcription (Source Text): An automated speech-to-text service was used to transcribe interview audio recordings, so it is expected that transcription errors and confusion will arise when the speaker’s voice is not clear (Signal-to-noise ratio concerns) or when the target speaker does not enunciate with clarity, or speaks with a non-standard accent. (Note: this difficulty is faced equally by all investigators using the same dataset).
5. De-contextualized Speech: To ensure the query used only speech originating from the subject in question (and not the interviewer), audio segments corresponding to the interviewer were removed before speech-to-text transcription. Consequently, the semantic content of the transcriptions may appear fragmented or discontinuous due to the missing contextual information and may influence the LLM chatbots’ performance in predicting AD and CN.
6. Due to the accessibility of the Dementia Bank repository, the possibility of the training data for the LLM chatbots containing instances from ADReSSo dataset cannot be entirely ruled out. This could limit the generalizability of the findings.
7. Furthermore, it is important to delve into the longitudinal progression of speech samples, as this aspect holds potential in alerting healthcare professionals, to serve as an early indicator of cognitive decline. To facilitate this investigation, the acquisition of a language dataset comprising speech samples from individuals presenting with mild cognitive impairment (MCI) alongside knowledge of their outcomes after three years becomes essential; the timing is also important and could help in predicting progression of MCI to AD [48].

Despite the utility of LLM chatbots in AD detection, there exists a need for robust validation by including multiple databases corresponding to different languages and audio responses and their transcribed text corresponding to various cognitive screening tools such as the MMSE, Montreal Cognitive Assessment (MoCA), MiniCog, or Rey Auditory Verbal Learning Test (RAVLT) [44,49,50,51,52]. Unlike the independent prompts used in this investigation, the prediction performance can be further tweaked by employing multi-level dependent prompts to extract structured information from transcribed text.

When utilizing online LLM chatbot platforms in clinical settings, data security measures must be prioritized to protect patient information. Access to the backend of these platforms could potentially reveal sensitive details about the user, underscoring the importance of access control or transitioning to local versions of LLM chatbots within secure networks.

Finally, a word of caution—with the increasing popularity of LLM chatbots, there is a likelihood that the public will turn to these technologies for screening psychological and cognitive illnesses. It is crucial that these chatbots provide clear disclaimers about their limitations, stressing that their assessments do not replace professional medical advice. They should refer users to consult healthcare professionals for a thorough evaluation and personalized treatment plan. Additionally, chatbots can provide further information on local health resources and support groups, urging users to seek help from qualified professionals. Such an approach can enhance the overall utility and safety of LLM chatbots for health screening and support responsibility at the community level.

## 5. Conclusions

The three LLM chatbots surveyed demonstrate the ability to identify AD vs. CN, surpassing chance-level performance, albeit with varying degrees of accuracy and confidence. When positively identifying AD, Bard performed best with an 89% true-positive rate but tended to misidentify CN as AD, often with high confidence (low “Unsure” rates); when positively identifying CN, GPT-4 performed best in identifying this true-negative at 56%, but tended to adopt a more diplomatic stance (moderate “Unsure” rates). By leveraging the unique strengths of different LLM chatbots (in their current form, as available publicly), we evaluated the performance and suitability as a first level tool to screen for cognitive decline based on spontaneous speech. However, further refinement is still needed to ensure reliability and effectiveness of these models in real-world healthcare contexts.

## Figures and Tables

**Table 1:** Performance metrics of the three LLM chatbots for Query 1 and Query 2, focusing on predicting AD (top) and predicting CN (bottom), respectively. Highest average performance among three LLM chatbots, for each pair of queries, are colored orange.

**AD Predicted**	**GPT-3.5**		**GPT-4**		**Bard**		**Metrics Averaged (across respective LLM chatbot)**
**Performance metrics**	**Query 1**	**Query 2**	**Query 1**	**Query 2**	**Query 1**	**Query 2**	
Accuracy	0.46	0.58	0.35	0.41	0.58	0.49	(0.52 + 0.38 + 0.54)/3 = 0.48
Sensitivity	0.60	0.83	0.11	0.29	0.89	0.89	(0.72 + 0.20 + 0.89)/3 = 0.60
Specificity	0.38	0.36	0.95	0.73	0.32	0.15	(0.37 + 0.84 + 0.24)/3 = 0.48
Precision	0.51	0.58	0.80	0.59	0.60	0.57	(0.55 + 0.70 + 0.59)/3 = 0.61
F1 Score	0.55	0.68	0.20	0.38	0.71	0.70	(0.62 + 0.29 + 0.71)/3 = 0.54
Overall Mean	0.50	0.61	0.48	0.48	0.62	0.56	(0.56 + 0.48 + 0.59)/3 = 0.54
**CN Predicted**	**GPT-3.5**		**GPT-4**		**Bard**		**Metrics Averaged (across respective LLM chatbot)**
**Performance metrics**	**Query 1**	**Query 2**	**Query 1**	**Query 2**	**Query 1**	**Query 2**	
Accuracy	0.46	0.58	0.35	0.41	0.58	0.49	(0.52 + 0.38 + 0.54)/3 = 0.48
Sensitivity	0.33	0.33	0.58	0.53	0.28	0.11	(0.33 + 0.56 + 0.20)/3 = 0.36
Specificity	0.72	0.91	0.27	0.59	0.91	1.00	(0.82 + 0.43 + 0.96)/3 = 0.73
Precision	0.60	0.80	0.66	0.73	0.77	1.00	(0.70 + 0.70 + 0.89)/3 = 0.76
F1 Score	0.43	0.47	0.62	0.61	0.41	0.20	(0.45 + 0.62 + 0.31)/3 = 0.46
Overall Mean	0.51	0.62	0.50	0.57	0.59	0.56	(0.57 + 0.54 + 0.58)/3 = 0.56

## Data Availability

The transcription data can be made available upon request from corresponding authors (balamurali_bt@sutd.edu.sg, jerming_chen@sutd.edu.sg).

## References

[1] Brodaty H., Donkin M. (2009). Family Caregivers of People with Dementia. Dialogues Clin. Neurosci..

[2] Brookmeyer R., Johnson E., Ziegler-Graham K., Arrighi H.M. (2007). Forecasting the Global Burden of Alzheimer’s Disease. Alzheimer’s Dement..

[3] Nandi A., Counts N., Chen S., Seligman B., Tortorice D., Vigo D., Bloom D.E. (2022). Global and Regional Projections of the Economic Burden of Alzheimer’s Disease and Related Dementias from 2019 to 2050: A Value of Statistical Life Approach. EClinicalMedicine.

[4] Livingston G., Huntley J., Sommerlad A., Ames D., Ballard C., Banerjee S., Brayne C., Burns A., Cohen-Mansfield J., Cooper C. (2020). Dementia Prevention, Intervention, and Care: 2020 Report of the Lancet Commission. Lancet.

[5] Banks R., Higgins C., Greene B.R., Jannati A., Gomes-Osman J., Tobyne S., Bates D., Pascual-Leone A. (2024). Clinical Classification of Memory and Cognitive Impairment with Multimodal Digital Biomarkers. Alzheimer’s Dement..

[6] Mintun M.A., Lo A.C., Duggan Evans C., Wessels A.M., Ardayfio P.A., Andersen S.W., Shcherbinin S., Sparks J., Sims J.R., Brys M. (2021). Donanemab in Early Alzheimer’s Disease. N. Engl. J. Med..

[7] van Dyck C.H., Swanson C.J., Aisen P., Bateman R.J., Chen C., Gee M., Kanekiyo M., Li D., Reyderman L., Cohen S. (2023). Lecanemab in Early Alzheimer’s Disease. N. Engl. J. Med..

[8] Blair M., Marczinski C.A., Davis-Faroque N., Kertesz A. (2007). A Longitudinal Study of Language Decline in Alzheimer’s Disease and Frontotemporal Dementia. J. Int. Neuropsychol. Soc..

[9] Meilán J.J.G., Martínez-Sánchez F., Carro J., Sánchez J.A., Pérez E. (2012). Acoustic Markers Associated with Impairment in Language Processing in Alzheimer’s Disease. Span. J. Psychol..

[10] Priyadarshinee P., Clarke C.J., Melechovsky J., Lin C.M.Y., B.T B., Chen J.-M. (2023). Alzheimer’s Dementia Speech (Audio vs. Text): Multi-Modal Machine Learning at High vs. Low Resolution. Appl. Sci..

[11] Rohanian M., Hough J., Purver M. (2021). Alzheimer’s Dementia Recognition Using Acoustic, Lexical, Disfluency and Speech Pause Features Robust to Noisy Inputs. arXiv.

[12] Qiao Y., Yin X., Wiechmann D., Kerz E. (2021). Alzheimer’s Disease Detection from Spontaneous Speech through Combining Linguistic Complexity and (Dis)Fluency Features with Pretrained Language Models. arXiv.

[13] Cintoli S., Favilli L., Morganti R., Siciliano G., Ceravolo R., Tognoni G. (2024). Verbal Fluency Patterns Associated with the Amnestic Conversion from Mild Cognitive Impairment to Dementia. Sci. Rep..

[14] Themistocleous C., Eckerström M., Kokkinakis D. (2020). Voice Quality and Speech Fluency Distinguish Individuals with Mild Cognitive Impairment from Healthy Controls. PLoS ONE.

[15] Yang Q., Li X., Ding X., Xu F., Ling Z. (2022). Deep Learning-Based Speech Analysis for Alzheimer’s Disease Detection: A Literature Review. Alz. Res. Ther..

[16] Pulido M.L.B., Hernández J.B.A., Ballester M.Á.F., González C.M.T., Mekyska J., Smékal Z. (2020). Alzheimer’s Disease and Automatic Speech Analysis: A Review. Expert Syst. Appl..

[17] Petti U., Baker S., Korhonen A. (2020). A Systematic Literature Review of Automatic Alzheimer’s Disease Detection from Speech and Language. J. Am. Med. Inform. Assoc..

[18] Amini S., Hao B., Zhang L., Song M., Gupta A., Karjadi C., Kolachalama V.B., Au R., Paschalidis I.C. (2022). Automated Detection of Mild Cognitive Impairment and Dementia from Voice Recordings: A Natural Language Processing Approach. Alzheimer’s Dement..

[19] Searle T., Ibrahim Z., Dobson R. (2020). Comparing Natural Language Processing Techniques for Alzheimer’s Dementia Prediction in Spontaneous Speech. arXiv.

[20] Syed Z.S., Syed M.S.S., Lech M., Pirogova E. (2021). Automated Recognition of Alzheimer’s Dementia Using Bag-of-Deep-Features and Model Ensembling. IEEE Access.

[21] Meghanani A., Anoop C.S., Ramakrishnan A.G. (2021). Recognition of Alzheimer’s Dementia from the Transcriptions of Spontaneous Speech Using fastText and CNN Models. Front. Comput. Sci..

[22] Yeung A., Iaboni A., Rochon E., Lavoie M., Santiago C., Yancheva M., Novikova J., Xu M., Robin J., Kaufman L.D. (2021). Correlating Natural Language Processing and Automated Speech Analysis with Clinician Assessment to Quantify Speech-Language Changes in Mild Cognitive Impairment and Alzheimer’s Dementia. Alz. Res. Therapy.

[23] Shah Z., Sawalha J., Tasnim M., Qi S., Stroulia E., Greiner R. (2021). Learning Language and Acoustic Models for Identifying Alzheimer’s Dementia from Speech. Front. Comput. Sci..

[24] Ying Y., Yang T., Zhou H. (2023). Multimodal Fusion for Alzheimer’s Disease Recognition. Appl. Intell..

[25] Biswas S.S. (2023). Role of Chat GPT in Public Health. Ann. Biomed. Eng..

[26] Lee P., Bubeck S., Petro J. (2023). Benefits, Limits, and Risks of GPT-4 as an AI Chatbot for Medicine. N. Engl. J. Med..

[27] Gellert G.A., Jaszczak J. (2023). Cardiovascular Disease Prevention Recommendations from an Online Chat-Based AI Model. JAMA.

[28] Pappagari R., Cho J., Joshi S., Moro-Velázquez L., Żelasko P., Villalba J., Dehak N. Automatic Detection and Assessment of Alzheimer Disease Using Speech and Language Technologies in Low-Resource Scenarios. Proceedings of the Interspeech 2021 ISCA.

[29] Pan Y., Mirheidari B., Harris J.M., Thompson J.C., Jones M., Snowden J.S., Blackburn D., Christensen H. Using the Outputs of Different Automatic Speech Recognition Paradigms for Acoustic- and BERT-Based Alzheimer’s Dementia Detection through Spontaneous Speech. Proceedings of the Interspeech 2021 ISCA.

[30] Wang W., Zheng V.W., Yu H., Miao C. (2019). A Survey of Zero-Shot Learning: Settings, Methods, and Applications. ACM Trans. Intell. Syst. Technol..

[31] Kojima T., Gu S.S., Reid M., Matsuo Y., Iwasawa Y. (2022). Large Language Models Are Zero-Shot Reasoners. arXiv.

[32] Wang N., Cao Y., Hao S., Shao Z., Subbalakshmi K.P. Modular Multi-Modal Attention Network for Alzheimer’s Disease Detection Using Patient Audio and Language Data. Proceedings of the Interspeech 2021 ISCA.

[33] Gauder L., Pepino L., Ferrer L., Riera P. Alzheimer Disease Recognition Using Speech-Based Embeddings From Pre-Trained Models. Proceedings of the Interspeech 2021 ISCA.

[34] Zhu Y., Obyat A., Liang X., Batsis J.A., Roth R.M. WavBERT: Exploiting Semantic and Non-Semantic Speech Using Wav2vec and BERT for Dementia Detection. Proceedings of the Interspeech 2021 ISCA.

[35] OpenAI ChatGPT, Mar 14 Version. Large Language Model. https://chat.openai.com/chat.

[36] Google Bard, May 10 Version. Large Language Model. https://bard.google.com/.

[37] Sarawagi S. (2007). Information Extraction. FNT Databases.

[38] Wei X., Cui X., Cheng N., Wang X., Zhang X., Huang S., Xie P., Xu J., Chen Y., Zhang M. (2023). Zero-Shot Information Extraction via Chatting with ChatGPT. arXiv.

[39] Luz S., Haider F., Fuente S.D.L., Fromm D., MacWhinney B. Detecting Cognitive Decline Using Speech Only: The ADReSSo Challenge. Proceedings of the Interspeech 2021 ISCA.

[40] Goodglass H., Kaplan E., Sandra W. (2001). BDAE: The Boston Diagnostic Aphasia Examination.

[41] Otter AI. https://otter.ai/.

[42] Temperature Check: A Guide to the Best ChatGPT Feature You’re (Probably) Not Using|LinkedIn. https://www.linkedin.com/pulse/temperature-check-guide-best-chatgpt-feature-youre-using-berkowitz/.

[43] Klimova B., Maresova P., Valis M., Hort J., Kuca K. (2015). Alzheimer’s Disease and Language Impairments: Social Intervention and Medical Treatment. Clin. Interv. Aging.

[44] Arevalo-Rodriguez I., Smailagic N., Roqué-Figuls M., Ciapponi A., Sanchez-Perez E., Giannakou A., Pedraza O.L., Bonfill Cosp X., Cullum S. (2021). Mini-Mental State Examination (MMSE) for the Early Detection of Dementia in People with Mild Cognitive Impairment (MCI). Cochrane Database Syst. Rev..

[45] Tombaugh T.N., McIntyre N.J. (1992). The Mini-Mental State Examination: A Comprehensive Review. J. Am. Geriatr. Soc..

[46] Crum R.M. (1993). Population-Based Norms for the Mini-Mental State Examination by Age and Educational Level. JAMA.

[47] Jin Z., Lu W. (2023). Tab-CoT: Zero-Shot Tabular Chain of Thought. arXiv.

[48] Moustafa A.A., Tindle R., Alashwal H., Diallo T.M.O. (2021). A Longitudinal Study Using Latent Curve Models of Groups with Mild Cognitive Impairment and Alzheimer’s Disease. J. Neurosci. Methods.

[49] Hoops S., Nazem S., Siderowf A.D., Duda J.E., Xie S.X., Stern M.B., Weintraub D. (2009). Validity of the MoCA and MMSE in the Detection of MCI and Dementia in Parkinson Disease. Neurology.

[50] Nasreddine Z.S., Phillips N.A., Bédirian V., Charbonneau S., Whitehead V., Collin I., Cummings J.L., Chertkow H. (2005). The Montreal Cognitive Assessment, MoCA: A Brief Screening Tool for Mild Cognitive Impairment. J. Am. Geriatr. Soc..

[51] Borson S., Scanlan J.M., Chen P., Ganguli M. (2003). The Mini-Cog as a Screen for Dementia: Validation in a Population-Based Sample. J. Am. Geriatr. Soc..

[52] Ricci M., Graef S., Blundo C., Miller L.A. (2012). Using the Rey Auditory Verbal Learning Test (RAVLT) to Differentiate Alzheimer’s Dementia and Behavioural Variant Fronto-Temporal Dementia. Clin. Neuropsychol..

